# The influence of body image on surgical decisions in adolescent idiopathic scoliosis patients

**DOI:** 10.6061/clinics/2017(03)01

**Published:** 2017-03

**Authors:** Paulo Alvim Borges, José Thomé de Carvalho Neto, Olavo Biraghi Letaif, Raphael Martus Marcon, Alexandre Fogaça Cristante

**Affiliations:** Hospital das Clínicas da Faculdade de Medicina da Universidade de São Paulo (IOT-HCFMUSP), Instituto de Ortopedia e Traumatologia, Laboratório de Investigação Médica, Divisão de Cirurgia da Coluna, São Paulo/SP, Brazil

**Keywords:** Scoliosis, Adolescent, Spinal Curvatures, Operative Surgical, Clinical Decision Support Systems, Decision-Making, Patient Participation

## Abstract

**OBJECTIVES:**

The objective of this study was to evaluate whether the severity of deformities in patients with adolescent idiopathic scoliosis contributes to patients’ decision regarding whether to undergo an operation.

**METHODS:**

We evaluated body image factors in adolescent idiopathic scoliosis patients. We evaluated the magnitude of the main scoliotic curve, gibbosity (magnitude and location), shoulder height asymmetry and patient’s age. We analyzed the correlation of these data with the number of years the patient was willing to trade for surgery, as measured by the time-trade-off method.

**RESULTS:**

A total of 52 patients were studied. We did not find a correlation between any of the parameters that were studied and the number of years that the patient would trade for the surgery.

**CONCLUSIONS:**

The magnitude of body deformities in patients with adolescent idiopathic scoliosis does not interfere with the decision to undertake surgical treatment.

## INTRODUCTION

A controversial topic for patients with adolescent idiopathic scoliosis (AIS) is whether they should undergo surgery 1. The literature defends indications for surgery that are based on the risk of scoliosis curve progression, the patient’s growth potential and the magnitude of the curve 2-11. The presence of progressive spinal deformity has a significant impact on the quality of life of patients with AIS who are treated without surgery 11-15.

According to Weiss et al., “In order to promote an intervention for a specific condition, it must be demonstrated that 1) the natural history of the condition is undesirable, 2) the intervention alters this natural history in a favorable and reproducible manner, 3) the complications are minimal, and 4) the long term side effects of the intervention are not detrimental, so that the risk-benefit ratio favors the intervention over the condition’s natural history.” Part of the decision of whether to operate remains an emotive choice among physicians, patients and their families 1.

There is evidence that surgical treatment for AIS is beneficial and reproducible, has controllable complications and is not deleterious 16-23. Recent data also indicate that surgical treatment of AIS improves patient body image acceptance 24. Therefore, to determine whether the risk-benefit ratio favors surgical treatment, it remains to be determined just how undesirable the condition of having AIS is to the patients who choose to undergo an operation.

It appears intuitive that body image perception contributes to the patient’s decision regarding their type of treatment, but we do not believe that this is as clear as it seems. Is it possible that the severity of deformities in patients with AIS causes them to prefer surgical treatment?

The objective of this study was to evaluate whether the severity of deformities in patients with AIS contributes to the patients’ decisions to undergo an operation.

## METHODS

In this study we evaluated body image factors that could contribute to the decision-making process for AIS treatment, whether surgical or nonsurgical. For this purpose, we used the time-trade-off (TTO) method 11. We placed the study subject in a bargaining situation in which he or she trades a subjective sensation of choice, of his or her own volition, for years of life. The more life years the patient exchanges for the condition offered, the greater the subjective desire to achieve the offer. For example, a patient who would trade 30 life years for an operation supposedly wants this surgery more than a patient who would trade 15 years of life for the same surgery. The use of TTO methods for subjective evaluation of quality of life has already been studied at length in the literature and is used in many areas of medical research 25-28.

The study was conducted in a public hospital, a referral center for orthopedics, between August 2014 and August 2015. Patients were recruited based on a convenience sample of patients who had previously been cataloged in the Spine Group as those awaiting surgery for correction of AIS. The indication for corrective surgery for scoliosis took into account the magnitude and the growth potential of the scoliotic curve as described in the established literature 1. Patients on that list were only recruited if they met the following inclusion criteria:

- Age >13 years
- Idiopathic scoliosis
- Informed consent form signed by the patient or legal guardian (in the case of minors)

Patients who met the following criteria were excluded from the study:

- Prior surgical procedure (any)
- Delayed neuropsychomotor development
- Illiteracy or being put back a grade at school
- Failure to attend the research interview

Recruitment was conducted over the phone or by conventional or electronic mail and was performed by a professional from the hospital who was blinded to the study evaluations.

The scientific study interview was made up of two parts and was performed by different professionals who were trained for this purpose. The professional who was responsible for the second part was also blinded to the study results. The first part consisted of an explanation about the scientific study provided to the patient and signing of the informed consent form. This explanation included a full discussion of the routine surgical risks and the possible outcomes of the surgical correction, with no case-by-case analysis of details, to standardize the explanations. The second part was the actual gathering of data, which was performed by spinal surgeons.

The following data were collected for the study:

1. **The magnitude of the main curve:** measured by the Cobb method 29 in degrees using standardized panoramic radiography in the hospital and comprising imaging from the occiput to the femoral heads, in the posteroanterior view, using the iSite Phillips measuring tool (Phillips. Blumenau, Santa Catarina, Brazil). This method has been previously validated 30.
2. **The magnitude of the dorsal gibbosity:** measured with a scoliometer 31 in degrees, as described with the Adam’s forward bend test 32.
3. **The location of the dorsal gibbosity:** “high hump”, when located in the upper half of the ribcage, and “low hump”, when located in the lower half of the ribcage.
4. **Shoulder imbalance (SI):** measured in centimeters. This distance was measured perpendicularly from the ground to the acromioclavicular (AC) joint bilaterally. Thus it is possible to have even (difference between shoulders ≤0.5 cm) or uneven (difference of >0.5 cm) shoulders.
5. **Age:** Measured in years.
6. **Life years that the patient would trade for scoliosis correction surgery.** According to the TTO method 33, the number of years that the patient would trade for corrective surgery was measured on a continuous scale with five-year intervals. At the time of this data collection, it was explained to the patients that they were being placed in a hypothetical situation but were supposed to answer with the belief that the situation was real. Colloquial language was used to ensure a good understanding of the dialogue. First, the patient was placed in the hypothetical situation that without surgery they would die at the age of 70 (an arbitrary number based on the average life expectancy of Brazilians). Subsequently, the patient was placed in a situation in which if he or she underwent an operation, he or she would die at age 65 rather than 70. The patient was then asked whether under these conditions, he/she continued to agree with the intervention. If so, a new condition was imposed, sequentially subtracting five life years, until the patient ceased to agree with the offer. The number of years that terminated the sequence subtracted from 70 indicated the result of the TTO.

All of the data were analyzed by a statistician who was also blinded to the study. The correlation between TTO and the remaining data (magnitude of the main curve, type of curve, SI, age, and magnitude and location of the dorsal gibbosity) were evaluated.

The distribution of continuous data was evaluated using the Shapiro-Wilk test and subjectively characterized via an analysis of histograms (distribution graphs). The continuous data were described by the mean and its respective standard deviation; categorical data were described by their absolute number and their respective percentage.

The Mann-Whitney test was used to test the degree of scoliosis curvature in 2 groups of data. In addition, some correlations were tested using Pearson’s correlation coefficient. *p*<0.05 was adopted for a statistically significant difference. The data were analyzed using version 22 of the SPSS software package for Mac.

## RESULTS

During the study period, 70 patients were treated and recruited for the study.

Eighteen of these patients did not attend the assessment interview. The remaining 52 patients who agreed to participate met all of the inclusion criteria. All of the evaluated patients were female. Clinical and anatomical characteristics of these patients are described in Tables 1 and 2.

The number of life years that the 52 patients would agree to trade for the surgery (TTO) averaged 37.25, with a standard deviation of 27.54 years.

**Figure 1:**
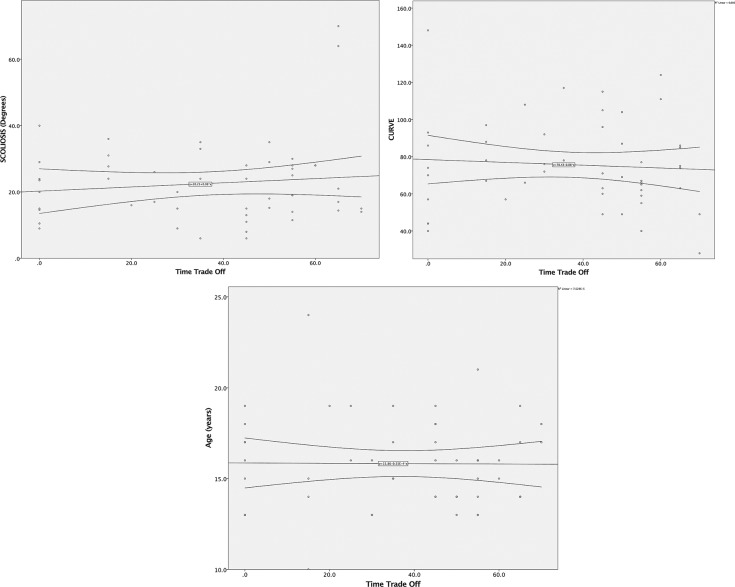
Pearson’s correlation tests show totally random distributions (from left to right: main curve, magnitude of gibbosity and age).

The continuous scale data, such as the magnitude of the main curve, the magnitude of gibbosity and age were analyzed with Pearson’s correlation test, and no correlation was found between the data and the TTO (*p*-values=0.617; 0.417 and 0.952, respectively; Figure 1).

Dichotomous data such as SI and the location of the dorsal gibbosity were analyzed using the Mann-Whitney test, and there was no significant difference between groups (*p*-values=0.789 and 0.507, respectively) in terms of the TTO.

## DISCUSSION

The magnitude of the main curve, the magnitude and location of the gibbosity and shoulder asymmetry are factors that esthetically alter body image and may possibly influence the treatment decision of patients with AIS. Age could also influence the decision because body image perception, expectations and life planning change as people grow older. However, in this study, no association was found between age or anatomical characteristics and the number of years that the patients would trade for surgery. Contrary to our initial intuition, the results demonstrate that these factors did not interfere with the decision to pursue surgical treatment, i.e., the size of the deformity did not influence the choice for surgery in this group of patients.

Although the proportion of women with AIS is much higher, according to the epidemiology 2, there could be differences between men and women in terms of the influence of body image factors. However, it was not possible to assess the proportions of this influence because all of the patients who were included in this study were female, although there was no restriction on gender at baseline.

Given the small sample size of our study, we could potentially be encountering a type II error due to a lack of power in our sample. The distribution shown in the correlation curves, however, is totally random and does not follow any trend, which leads us to believe that type II error is unlikely.

There may be no factors that are associated with body image that influence decision-making. It is possible that patients consider other types of factors when opting for surgery; perhaps patients consider their social acceptance in general, not body image changes alone.

The choice to undergo surgery to treat AIS is highly complex and multifactorial, and this study did not assess all of the possible variables that are involved in the decision. One example of a possible factor that was not studied in women was the symmetry of the body contours (such as the iliac crest height and breast dimensions). There may also be as-yet-unknown factors that contribute to the decision to undergo an operation. Ethnic and cultural factors among populations may strongly influence body image and alter the weight of the factors to be evaluated.

The realization that possible differences in the magnitude of deformities in scoliosis do not influence the patients’ decisions whether to operate can be significantly helpful for attending physicians when they are guiding decisions together with patients and their families. For patients who insist on aesthetics and body image as a justification to undergo the operation, we can contemplate the existence of a possible secondary gain or a reason that is not revealed by the patient to undergo the operation because our results indicate that the severity of deformities does not interfere with the decision. In this manner, physicians can treat the surgical indication with more caution.

The magnitude of body deformities in patients with AIS does not interfere with the decision to undergo surgical treatment.

## AUTHOR CONTRIBUTIONS

The manuscript was produced, reviewed and approved by all of the authors collectively. Borges PA designed the study, collected data, interpreted the results, wrote the manuscript and approved the manuscript final version to be published. Carvalho Neto JT designed the study, collected data, interpreted the results, wrote the manuscript and approved the final version of the manuscript to be published. Letaif OB designed the study, collected data, interpreted the results, wrote the manuscript and approved the final version of the manuscript to be published. Marcon RM designed the study, collected data, interpreted the results, wrote the manuscript and approved the final version of the manuscript to be published. Cristante AF designed the study, collected data, interpreted the results, wrote the manuscript and approved the final version of the manuscript to be published.

## Figures and Tables

**Table 1:** Descriptive statistics of the continuous data of the patients who were included in the study.

Data	Mean	Standard deviation
Age (years)	15.82	2.47
Gibbosity (degrees)	22.48	12.24
Main curve (degrees)	75.46	23.66

**Table 2:** Descriptive statistics of the dichotomous data of the patients who were included in the study.

Shoulder	Frequency	Percentage
Symmetrical	18	34.6
Asymmetrical	34	65.4
Total	52	100.0

Hump	Frequency	Percentage
High	25	48.1
Low	27	51.9
Total	52	100.0
